# Functional Gene-Set Analysis Does Not Support a Major Role for Synaptic Function in Attention Deficit/Hyperactivity Disorder (ADHD)

**DOI:** 10.3390/genes5030604

**Published:** 2014-07-22

**Authors:** Anke R. Hammerschlag, Tinca J. C. Polderman, Christiaan de Leeuw, Henning Tiemeier, Tonya White, August B. Smit, Matthijs Verhage, Danielle Posthuma

**Affiliations:** 1 Department of Complex Trait Genetics, Center for Neurogenomics and Cognitive Research, Neuroscience Campus Amsterdam, VU University Amsterdam, De Boelelaan 1085, 1081 HV Amsterdam, The Netherlands; E-Mails: a.r.hammerschlag@vu.nl (A.R.H.); tinca.polderman@vu.nl (T.J.C.P.); c.deleeuw@science.ru.nl (C.L.); 2 Institute for Computing and Information Sciences, Radboud University Nijmegen, P.O. Box 9010, 6500 GL Nijmegen, The Netherlands; 3 Department of Child and Adolescent Psychiatry, Erasmus University Medical Center and Sophia Children’s Hospital, P.O. Box 2060, 3000 CB Rotterdam, The Netherlands; E-Mails: h.tiemeier@erasmusmc.nl (H.T.); t.white@erasmusmc.nl (T.W.); 4 Department of Molecular and Cellular Neurobiology, Center for Neurogenomics and Cognitive Research, Neuroscience Campus Amsterdam, VU University Amsterdam, De Boelelaan 1085, 1081 HV Amsterdam, The Netherlands; E-Mail: guus.smit@vu.nl; 5 Department of Functional Genomics, Center for Neurogenomics and Cognitive Research, Neuroscience Campus Amsterdam, VU University Amsterdam, De Boelelaan 1085, 1081 HV Amsterdam, The Netherlands; E-Mail: matthijs.verhage@cncr.vu.nl; 6 Department of Clinical Genetics, VU University Medical Center, P.O. Box 7057, 1007 MB Amsterdam, The Netherlands

**Keywords:** complex trait, polygenic, gene network, biological pathway, synapse, GWAS, PGC

## Abstract

Attention Deficit/Hyperactivity Disorder (ADHD) is one of the most common childhood-onset neuropsychiatric disorders. Despite high heritability estimates, genome-wide association studies (GWAS) have failed to find significant genetic associations, likely due to the polygenic character of ADHD. Nevertheless, genetic studies suggested the involvement of several processes important for synaptic function. Therefore, we applied a functional gene-set analysis to formally test whether synaptic functions are associated with ADHD. Gene-set analysis tests the joint effect of multiple genetic variants in groups of functionally related genes. This method provides increased statistical power compared to conventional GWAS. We used data from the Psychiatric Genomics Consortium including 896 ADHD cases and 2455 controls, and 2064 parent-affected offspring trios, providing sufficient statistical power to detect gene sets representing a genotype relative risk of at least 1.17. Although all synaptic genes together showed a significant association with ADHD, this association was not stronger than that of randomly generated gene sets matched for same number of genes. Further analyses showed no association of specific synaptic function categories with ADHD after correction for multiple testing. Given current sample size and gene sets based on current knowledge of genes related to synaptic function, our results do not support a major role for common genetic variants in synaptic genes in the etiology of ADHD.

## 1. Introduction

Attention Deficit/Hyperactivity Disorder (ADHD) is one of the most common childhood-onset neuropsychiatric disorders. The worldwide prevalence is estimated at ~5% [1], and remained relatively stable across the last three decades [2]. ADHD is characterized by a persistent pattern of inattention and/or impulsiveness and hyperactivity. Despite high heritability estimates for ADHD, averaging 70% [3], the identification of genes has been difficult. Most likely this is mainly due to the polygenic character of ADHD, similar to that of other complex traits, meaning that many genetic variants with small effects contribute to ADHD risk [4].

Genome-wide association studies (GWAS) of ADHD have yielded no significant single nucleotide polymorphism (SNP) associations thus far [5]. However, it has been reported that the top hits of GWAS point to the involvement of synaptic processes such as neurotransmission, cell-cell communication systems, potassium channel subunits and regulators, and more basic processes like neuronal migration, neurite outgrowth, spine formation, neuronal plasticity, cell division, and adhesion [6,7,8]. Furthermore, many genes previously implicated in ADHD [9] are expressed in the synapse (*i.e.*, *DBH*, *SLC6A2*, *ADRA2A*, *HTR1B*, *HTR2A*, *TPH1/2*, *MAOA*, *CHRNA4*, *SNAP25*, and *BDNF*), suggesting the involvement of synaptic function in the etiology of ADHD.

In addition to common genetic variants, rare variants may contribute to ADHD risk. Increased structural variation burden has been reported, particularly in subjects with intellectual disability [10,11,12,13]. Interestingly, biological pathways enriched for GWAS SNP associations with low *p*-values overlap with pathways enriched for rare structural variants, including pathways important for synaptic function [12]. Of special interest are SNPs and duplications spanning the *CHRNA7* gene, which is primarily involved in modulation of rapid synaptic transmission and which has been associated with other neuropsychiatric phenotypes in addition to ADHD [12,13]. Furthermore, strong associations have been reported for structural variation affecting metabotropic glutamate receptor genes and genes that interact with them. Several of these genes are important modulators of synaptic transmission and neurogenesis [11].

Given the polygenic nature of ADHD, it is likely that non-random combinations of genetic variants are involved in the etiology of ADHD. Genes do not work in isolation; rather, they form complex molecular networks and cellular pathways. Therefore, it is plausible that the numerous genetic variants of small effect aggregate in genes that share a similar cellular function. Evaluating the joint effect of multiple SNPs in functionally related genes increases the statistical power to detect associations with ADHD compared to single SNP methods, as it reduces multiple testing. Moreover, single SNP associations do not necessarily lead to knowledge about underlying biological mechanisms, while a set of genes with the same function could result in more insight in the molecular or cellular mechanisms of ADHD [14].

Prior studies that tested the joint effect of genetic variants generally grouped genes based on biological pathways. However, grouping genes based on cellular function (“horizontal grouping”) instead of biological pathways (“vertical grouping”) may be especially powerful in synaptic protein networks [15,16]. Many different pathways regulate synaptic function, but act not independent, as many proteins act across pathways. For example, different neuromodulator pathways (e.g., dopamine or serotonin) include receptors that are activated by the specific neuromodulators, but are functionally and often structurally similar to each other. It may well be that genetic variants influencing complex traits like ADHD concentrate at similar cellular function, by which they influence different pathways leading to similar consequences in synaptic function.

The majority of gene-set analyses that have been conducted have used publicly available gene sets. However, currently available public gene sets are generally incomplete and neither error-free nor unbiased, especially with regard to genes active in the brain [17,18]. Fortunately, expert-curated sets of genes are increasingly becoming available, such as the mir-137 gene set [19], specific synaptic gene sets [15], and gene sets for glial function [20].

As the results of previous GWAS and genes affected by structural variation suggested involvement of synaptic function, we hypothesized that synaptic processes play a role in the etiology of ADHD. Collective testing of genetic variants in genes grouped according to similar synaptic functions may be the most optimal way to test this. Therefore, we applied a functional gene-set analysis for ADHD using 18 previously published, expert-curated pre- and postsynaptic gene sets [15]. To our knowledge, this is the first study to conduct hypothesis-driven gene-set analysis for ADHD by grouping synaptic genes according to cellular function. We used ADHD data from the Psychiatric Genomics Consortium (PGC) [5].

## 2. Methods

### 2.1. Sample

We used GWAS summary statistics from the currently largest publicly available ADHD data set, as provided by the PGC [5]. Details on the data set have been described previously [5]. In short, the data set consisted of four projects: the Children’s Hospital of Philadelphia (CHOP), phase I of the International Multicenter ADHD Genetics Project (IMAGE), phase II of IMAGE (IMAGE II), and the Pfizer-funded study from the University of California, Los Angeles, Washington University, and Massachusetts General Hospital (PUWMa). The total sample consisted of 896 unrelated cases and 2455 controls, and 2064 trio samples (alleles transmitted to offspring were considered as “trio cases”, and non-transmitted alleles as “pseudo-controls”). All samples were of European ancestry and met diagnostic criteria for ADHD as defined by the DSM-IV. All samples underwent the same quality control and analysis steps. The strongest single SNP association with ADHD in this data set was *p* = 1.10 × 10^−6^ [5].

### 2.2. Defining Functional Gene Sets

Generation of the synaptic gene sets has been described previously [15]. Briefly, synaptic gene grouping was based on cellular function as determined by previous synaptic protein identification experiments and data mining for synaptic genes and gene function. This resulted in the inclusion of 1028 genes, expressed in either the pre- or postsynapse or in both, divided over 17 synaptic gene sets with a specific synaptic function, and one synaptic gene set with unassigned cellular function. The gene sets with gene IDs are available at the Complex Trait Genetics webpage [21].

### 2.3. Power Analysis

The Genetic Power Calculator (GPC) [22,23] was used to define the minimal genotype relative risk that could reliably be detected for a gene set given the current sample size. Because the PGC data set consists of both case-control samples and trio samples, power was calculated using the weighted mean of the noncentrality parameters of the samples. To use the GPC for gene-set analysis, we assumed that the risk allele frequency represents the average allele frequency of all contributing risk variants in a gene set, and that the relative risk is representing the global effect of the gene set. We further used a disease prevalence of 5% (as estimated by Polanczyk *et al*. [1]), and a multiplicative model (power calculation based on the allelic test). Tests were corrected for the number of gene sets (α = 0.05/18 = 2.8 × 10^−3^).

### 2.4. Gene-Set Analysis

Gene-set analysis was conducted using JAG [24]. To test the hypothesis that synaptic function was associated with ADHD, we conducted self-contained tests for each gene set and one overall test including all synaptic gene sets. For each gene set, the test statistic was defined as the sum over the −log_10_ of SNP *p*-values annotated to genes in that gene set. These SNP *p*-values were taken from the PGC association results. To allow for unbiased interpretation of the test statistic, 10,000 permutations were conducted in which any relation between a genetic variant and affection status was disconnected. As such, linkage disequilibrium (LD), and number of SNPs and genes within each gene set stayed intact. For each permutation of the data set, the test statistics of the gene sets were computed. The self-contained *p*-value was calculated as the proportion of test statistics in the permuted data sets that was higher than the original test statistic. Bonferroni correction was applied to account for multiple testing with a corrected significance threshold of α = 0.05/18 = 2.8 × 10^−3^.

For the permutations of the data set, we used the genotype data of the European ancestry samples from the 1000 Genomes project [25] with a simulated binary phenotype (as we had no access to raw data of the PGC). Using this as reference data, we could appropriately account for LD effects on correlations in SNP *p*-values in the PGC association data. For the test statistics of the original gene sets, only SNPs that were also available in the 1000 Genomes genotype data were used.

Competitive tests were performed for gene sets found to be significant in the self-contained test. While self-contained tests evaluate whether a gene set is associated with ADHD under the null hypothesis of no association, a competitive test shows whether the observed (self-contained) association is stronger than expected by chance for gene sets with the same number of genes. To this end, 150 random gene sets were generated, matching for the same number of genes. JAG calculated a self-contained *p*-value for each of these random gene sets. The competitive *p*-value was then computed as the proportion of random gene sets with self-contained *p*-values lower than the self-contained *p*-value for the gene set itself. Only gene sets with a competitive *p*-value < 0.05 were considered to be significant.

## 3. Results

### 3.1. Power Analysis 

**Figure 1:**
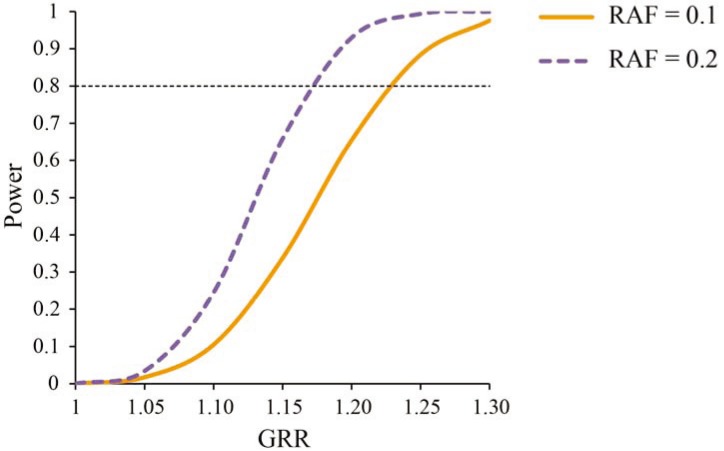
Statistical power to detect gene sets in the Psychiatric Genomics Consortium (PGC) Attention Deficit/Hyperactivity Disorder (ADHD) sample. Power is displayed for different genotype relative risks (GRR), and risk allele frequencies (RAF) of 0.1 and 0.2. The weighted mean of the noncentrality parameters of the case-control sample (896 cases and 2455 controls) and trio sample (2064 trios) was used to calculate power. Power analyses assume a disease prevalence of 5% and a multiplicative model. We assumed that gene sets behave as individual single nucleotide polymorphisms (SNPs). Tests are corrected for number of gene sets (α = 2.8 × 10^−3^). Dotted horizontal line represents power of 0.80.

Power analyses showed that for gene sets containing on average SNPs with a risk allele frequency (RAF) of at least 0.1, our sample had sufficient power (≥0.80) to detect gene sets with a genotype relative risk (GRR) of 1.23 (Figure 1). For gene sets containing a mean RAF of at least 0.2, we had sufficient power to detect gene sets with a GRR of 1.17.

### 3.2. Gene-Set Analysis 

A total number of 1,206,461 SNPs were available for gene-set analysis. Of these, 61,413 SNPs mapped to 956 genes (out of 1028) within our gene sets. All 956 synaptic genes together were significantly associated with ADHD in the self-contained test (Table 1). However, the competitive test showed that the synaptic genes were not more strongly associated with ADHD than randomly generated gene sets matched for same number of genes, suggesting that the self-contained *p*-value was significant merely due to a large number of SNPs being evaluated, which did not particularly aggregate in genes involved in synaptic function.

**Table 1:** Association findings between synaptic gene sets and ADHD.

Gene Set	Number of Genes in Original Set	Number of Genes Present in GWAS Data	Number of SNPs Present in GWAS Data	Self-Contained *p*-Value (α = 2.8 × 10^−3^)	Competitive *p*-Value (α = 0.05)
All synaptic genes	1028	956	61413	0.0393 *	0.1733
Ion balance/transport	43	40	1454	0.0118	NA
Cell metabolism	57	51	1059	0.0429	NA
Endocytosis	26	26	1075	0.0554	NA
Cell adhesion and trans-synaptic signaling	81	76	13550	0.0709	NA
Exocytosis	87	83	4855	0.0962	NA
Protein cluster	47	42	4182	0.1491	NA
Peptide/neurotrophin signals	28	25	1742	0.1659	NA
Structural plasticity	98	90	4655	0.1764	NA
Tyrosine kinase signaling	7	7	1281	0.2030	NA
Neurotransmitter metabolism	29	27	1059	0.2959	NA
RNA and protein synthesis, folding and breakdown	71	64	1152	0.4994	NA
Ligand-gated ion channel signaling	36	32	2935	0.6500	NA
G-protein-coupled receptor signaling	41	40	3129	0.6578	NA
Unassigned	61	53	2258	0.6644	NA
Intracellular signal transduction	150	145	9563	0.7001	NA
G-protein relay	27	25	946	0.7047	NA
Intracellular trafficking	80	75	2024	0.7334	NA
Excitability	59	56	4508	0.7914	NA

* α = 0.05.

Self-contained tests for the specific synaptic gene sets showed associations at nominal significance levels for the involvement of *ion balance/transport* and *cell metabolism* in ADHD (Table 1). However, these associations did not survive Bonferroni correction. All other self-contained *p*-values were >0.05. We thus conclude that no significant associations were found between any of the specific synaptic gene sets and ADHD. Consequently, no subsequent competitive tests were performed for the synaptic gene sets of specific functions.

## 4. Discussion

Results from previous GWAS have led to the conclusion that ADHD is a heritable, yet polygenic disorder influenced by many genetic variants of small effect. Top hits from previous studies have suggested a role for synaptic processes in the etiology of ADHD. In the current study, we tested the hypothesis that genetic variants that influence the risk for ADHD cluster in synaptic gene sets. We used expert-curated gene sets of pre- and postsynaptic genes. Using the largest public ADHD GWAS sample currently available, our study had sufficient statistical power to detect gene sets representing a GRR of at least 1.17 (or 1.23 for less common alleles) for the liability to develop ADHD. The self-contained test of all synaptic genes together showed a significant association with ADHD. However, for complex traits that are polygenic, any large group of genes is likely to be associated due to background polygenic effects. The competitive test showed that the association was not stronger compared to that of randomly generated gene sets with the same number of genes. This suggests that the association was not a result of the selection of synaptic genes, but merely because of the large number of genes. Hence, our results support the idea that ADHD is a polygenic disorder, and suggest that overall synaptic function does not play a major role in the etiology of ADHD, given current synaptic genes.

In addition, no specific synaptic function categories were associated with ADHD after correction for multiple testing. These results suggest that if common genetic variants in the current synaptic gene sets with a specific function play a role in the etiology of ADHD, their effect is modest at most, even when considering the joint effect of multiple genetic variants.

Although previous analyses suggested involvement of several synaptic processes in ADHD [6,7,11,12,13], it should be kept in mind that the majority of previous results reported non-significant, suggestive results, and hence no strong conclusions could be drawn regarding the impact of those processes on ADHD. For example, a recent study used a different type of categorization of gene sets: they constructed gene sets based on pathways and candidate genes, and did report significant associations of dopamine/norepinephrine and serotonin pathways, and genes involved in neuritic outgrowth, with the hyperactive/impulsive component of ADHD [26]. However, in this study competitive tests to investigate if reported associations were stronger than can be expected by the polygenic nature of ADHD were not performed. Consequently, it remains unclear whether the reported associations are due to the background polygenic effects like our apparent association of synaptic genes with ADHD.

Synaptic function has been implicated and confirmed for other psychiatric disorders, especially schizophrenia [19,24] and bipolar disorder [27,28]. For example, gene sets of *cell adhesion and trans-synaptic signaling* and *excitability* showed replicated associations with schizophrenia [19,24]. Recent cross-disorder analyses by the PGC reported overlap in genetic liability between psychiatric disorders (schizophrenia, bipolar disorder, major depressive disorder, autism spectrum disorder, and ADHD) [29,30]. However, of all five psychiatric disorders, ADHD showed the weakest genetic overlap with other psychiatric disorders, having only a moderate genetic correlation with major depressive disorder, and showing no overlap with schizophrenia, bipolar disorder, and autism spectrum disorder. Our current findings fit into this overall picture of a separate genetic etiology of ADHD, by showing no evidence for an association with common variants in the current curated list of synaptic genes.

The list of genes involved in synaptic function is however a dynamic list: it depends on available experimental data and expert curation. When more experimental data is generated more genes may be included, which may have been missed in the current analyses. However, if genetic variants with an effect on ADHD risk aggregate in genes that are active in the synapse, it is expected that many genes within this gene set play a role in ADHD. Thus, an indication of association should be present if any of our current gene sets has a strong effect on ADHD risk, even when the current gene sets are not complete. Our results do not show any clear trends of association between the gene sets and ADHD. 

An alternative explanation for the lack of association in our study could be the heterogeneous nature of ADHD. It is known that ADHD is characterized by a heterogeneous manifestation of symptoms, possibly reflecting genetic heterogeneity [31]. Genetic heterogeneity makes it more challenging to detect genetic variation that plays a role in the etiology of ADHD, as the heterogeneity results in an apparent reduction of the effect sizes of true genetic variants. The current lack of association of synaptic functions with ADHD diagnosis together with previous reports that implicate a role of synaptic function based on smaller scaled samples, may reflect the involvement of synaptic function in only very specific sub-populations of ADHD symptoms. Future studies focusing on ADHD symptom profiles are needed to detect such specific associations between synaptic function and ADHD subtypes.

## 5. Conclusions

We find no evidence for involvement of specific synaptic functions in the etiology of ADHD, given current sample size and gene sets based on current knowledge of genes related to synaptic function. Our results suggest that if common genetic variants in the current synaptic gene sets play a role in the etiology of ADHD, their effect is modest at most, even when considering the joint effect of multiple genetic variants.
